# Biofeedback-Based Closed-Loop Phytoactuation in Vertical Farming and Controlled-Environment Agriculture

**DOI:** 10.3390/biomimetics9100640

**Published:** 2024-10-18

**Authors:** Serge Kernbach

**Affiliations:** CYBRES GmbH, Research Center of Advanced Robotics and Environmental Science, Melunerstr. 40, 70569 Stuttgart, Germany; serge.kernbach@cybertronica.de.com

**Keywords:** biohybrid systems, phytosensing, phytoactuation, impedance spectroscopy, electrochemical sensors, biofeedback, precision agriculture, pollution detection

## Abstract

This work focuses on biohybrid systems—plants with biosensors and actuating mechanisms that enhance the ability of biological organisms to control environmental parameters, to optimize growth conditions or to cope with stress factors. Biofeedback-based phytoactuation represents the next step of development in hydroponics, vertical farming and controlled-environment agriculture. The sensing part of the discussed approach uses (electro)physiological sensors. The hydrodynamics of fluid transport systems, estimated electrochemically, is compared with sap flow data provided by heat-based methods. In vivo impedance spectroscopy enables the discrimination of water, nutrient and photosynthates in the plant stem. Additionally to plant physiology, the system measures several air/soil and environmental parameters. The actuating part includes a multi-channel power module to control phytolight, irrigation, fertilization and air/water preparation. We demonstrate several tested in situ applications of a closed-loop control based on real-time biofeedback. In vertical farming, this is used to optimize energy and water consumption, reduce growth time and detect stress. Biofeedback was able to reduce the microgreen production cycle from 7 days to 4–5 days and the production of wheatgrass from 10 days to 7–8 days, and, in combination with biofeedback-based irrigation, a 30% increase in pea biomass was achieved. Its energy optimization can reach 25–30%. In environmental monitoring, the system performs the biological monitoring of environmental pollution (a low concentration of O_3_) with tomato and tobacco plants. In AI research, a complex exploration of biological organisms, and in particular the adaptation mechanisms of circadian clocks to changing environments, has been shown. This paper introduces a phytosensor system, describes its electrochemical measurements and discusses its tested applications.

## 1. Introduction

Biohybrid developments [1,2] combine technological and biological systems to advance their capabilities and to achieve a functionality that it is not possible for any of these systems to achieve separately. The principles of biohybrid systems were explored in swarm research of bees and swarm robots [3,4]; fish, bees and robots [5,6]; plants and actuated architecture [7]; plants and robots [8]; and plants and AI systems [9]. In this work, we focus on the practical aspects of biohybrid research, which has applications in environmental monitoring, precision agriculture and vertical farming.

The central element of a biohybrid architecture is a sensing part that interfaces biological and technological objects. This part represents key scientific and technological challenges—new types of sensors are in development based on electrochemical impedance spectroscopy (EIS) [10,11], nuclear magnetic resonance (NMR) imaging [12], aquaporin water channels [13] and spin-controlled physical chemistry [14,15]. In the presented development, we focus on electrochemical methods applied to the fluid transportation system in plant stems [16]. Fluid transportation in the phloem and xylem tissues is the main physiological mechanism for the distribution of water, nutrients and photosynthates throughout the plant [17]. State-of-the-art approaches to sap flow measurements are thermal methods such as heat balance [18] and heat pulse [19].

This paper describes a novel approach for phytosensing based on the hydrodynamic and ionic parameters of fluid transportation sensed with differential EIS. The water and sap movements in the stem create different degrees of fluid enrichment in plant tissues, which changes their dielectric properties, ionic flows and, finally, electrochemical impedances. By performing differential EIS in the lower and upper parts of the stem [20], it is possible to sense the temporal dynamics and spatial distribution of fluid movement. We developed a hydrodynamic model and tested it with light, irrigation and environmental stressors and compared it with thermal sap flow measurements. Different parameters of fluid transportation or circadian rhythms are detectable with this method. They are species-specific and follow internal metabolic activity that can be further optimized by light, irrigation or fertilization.

Since the chemical and ionic properties of upflow (water with nutrient from soil) and downflow (organic products from the photosynthesis) are different [15,21], performing time–frequency EIS enables us to distinguish both flows and characterize them. In several cases, the sap needs to be extracted from plants [22]; the detection of downflow allows for the optimizing of this process. Electrochemical characterization is performed in vivo in real time and is used in several phytosensing developments.

We demonstrate three in situ-tested applications based on real-time biofeedback using this approach. First of all, the electrochemical sensing of hydrodynamic parameters is used for stress detection; measurements were taken of the water deficit strategy and a biological detection of O_3_ pollution in environmental monitoring was also conducted. One of the main applications of this method is related to the closed-loop control of phytoactuators in various agricultural systems such as vertical farms, precision agriculture, controlled-environment agriculture or hydroponics. The tested biofeedback strategies are to optimize energy and water consumption and reduce the growth time via the optimization of spectral light and irrigation. In terms of AI research, we demonstrate a complex exploration of biological organisms, and in particular the adaptive mechanisms of circadian clocks [23,24]. The tested plant species include different microgreens (e.g., wheat and pea), productive plants (e.g., tomato and tobacco) and room plants such as dracena. Further, in this paper, a (phyto)sensing/actuating system is introduced in Section 2, electrochemical measurements are described in Section 3 and tested applications are discussed in Section 4.

## 2. Methods and Setup

**Figure 1:**
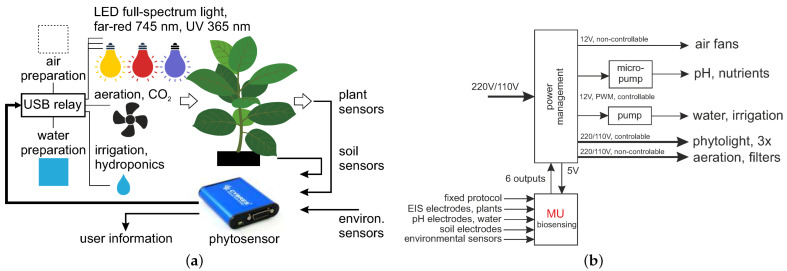
(**a**) General scheme of a closed-loop system with actuating and sensing parts. (**b**) The power management module used, with phytosensors.

The general scheme of the closed-loop system is shown in Figure 1a. It includes actuating (phytolight, aeration and irrigation) and sensing (plant physiology, soil and environment) parts, creating the closed-loop system. Air and water preparation represent the experimental features for testing different air/water pollutants and contaminants, adding fertilizers to the water, or enriching the air with CO_2_. The used phytosensing system [25] measures and records multiple physiological and environmental parameters; see Table 1.

The turning on/off of all actuators is accomplished with the power management module; see Figure 1b. The phytosensor can directly control up to six solid-state/electromechanical relays, high-power MOSFETs and other external switching devices. Such relays can be used for periodical irrigation and the control of phytolight or any other phytoactuators. Periodical timers execute tasks related to on/off switching at specified time points.

**Figure 2:**
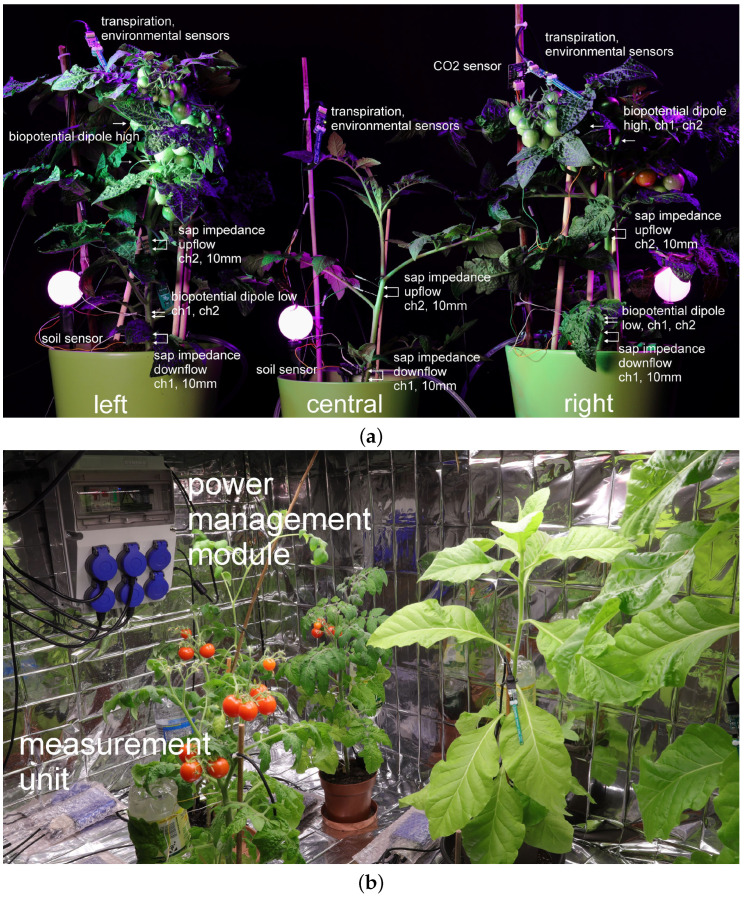
(**a**) Setup of three tomato plants used in experiments; different phytosensors are shown. (**b**) Setup of the power management module and tomato/tobacco plants.

Experiments have been conducted, with several setups, using tomato *solanum lypopersicum*, *dracena fragrans* and tobacco *nicotiana tabacum, var. Habana* plants, as well as microgreens (e.g., wheat and pea); see Figure 2. This selection of plant species was determined by seasonal availability, different plant morphologies (stem and leaves for sap flow measurements) and the usage of these plants (productive and room plants).

Measurements of electrochemical impedance were conducted with differential EIS in spectral and continuous-time modes, with Ag99 electrodes inserted in the upper and lower parts of stem tissues. These sensors are further denoted as the upper and lower EIS sensors. The distance between electrodes is selected experimentally so that the RMS impedance varies between 50 kOhm and 100 kOhm, typically at a distance of 10 mm. The penetration depth is about 2–3 mm to create stable mechanical contact and reach phloem and xylem tissue. In several experiments, electrodes were inserted directly into the leaf or completely penetrated the stem. Similar Ag99 electrodes were also used to measure biopotentials; however, they require a larger dipole than the impedance measurements.

**Figure 3:**
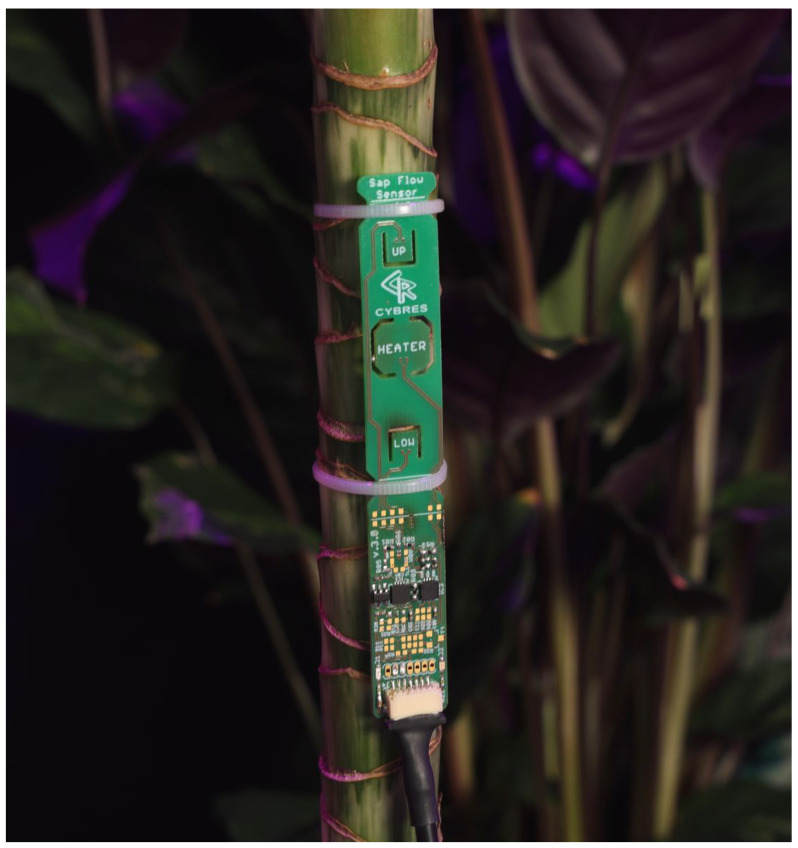
Heat-based sap flow sensor with two upflow $T_{U}$ and downflow $T_{D}$ temperature sensors and a heater between them.

The heat-based sap flow sensor is shown in Figure 3. It has two upflow and downflow temperature sensors and a thermo-stabilized heater between them. The sensor supports both heat balance and heat impulse measurements. The heater temperature is measured by an independent sensor; the PID controller monitors the heating dynamics with high accuracy. Embedded electronics form all necessary signals. Since the heat-based sap flow sensor requires a strong and thick stem, which is not present in some plant species (e.g., young tomato plants, microgreens, leafy greens, strawberries), most of the fluid measurements were conducted using EIS sensors.

EIS utilizes the principle of current measurements. The current loop systems are not susceptible to electromagnetic interferences and therefore do not require a Faraday cage (this principle is widely used in analog sensors, early telephony and remote control). To avoid the USB ground loops, phytosensors are connected to the PC via USB-to-USB isolators; this also reduces the level of noise. Since tissue impedance has a slower reaction time than action potentials or variation potentials, measurement cycles are typically 120 min for the background (without stimuli) and experimental regions. Tissue impedance in the stem and leaf areas depends on different parameters such as irrigation, temperature, humidity, cyclic light rhythms and others [26]. To create single-stimulus experiments, these parameters are fixed and do not vary across experimental attempts. The transpiration rate, leaf temperature, soil moisture/temperature, light intensity, air temperature/humidity and CO_2_ are also measured and recorded; see Table 1.

No chemicals or reagents were used during the experiments with pot plants. Hydroponic production uses a pH down solution with a pH controller (to maintain the pH at 6) and NPK fertilizer (6.4 + 4.5 + 6.7) in the initial preparation of the irrigation system (with no variations during the experiments).

## 3. Measurements

### 3.1. Heat-Based Sap Sensors for Long-Term Measurements

**Figure 4:**
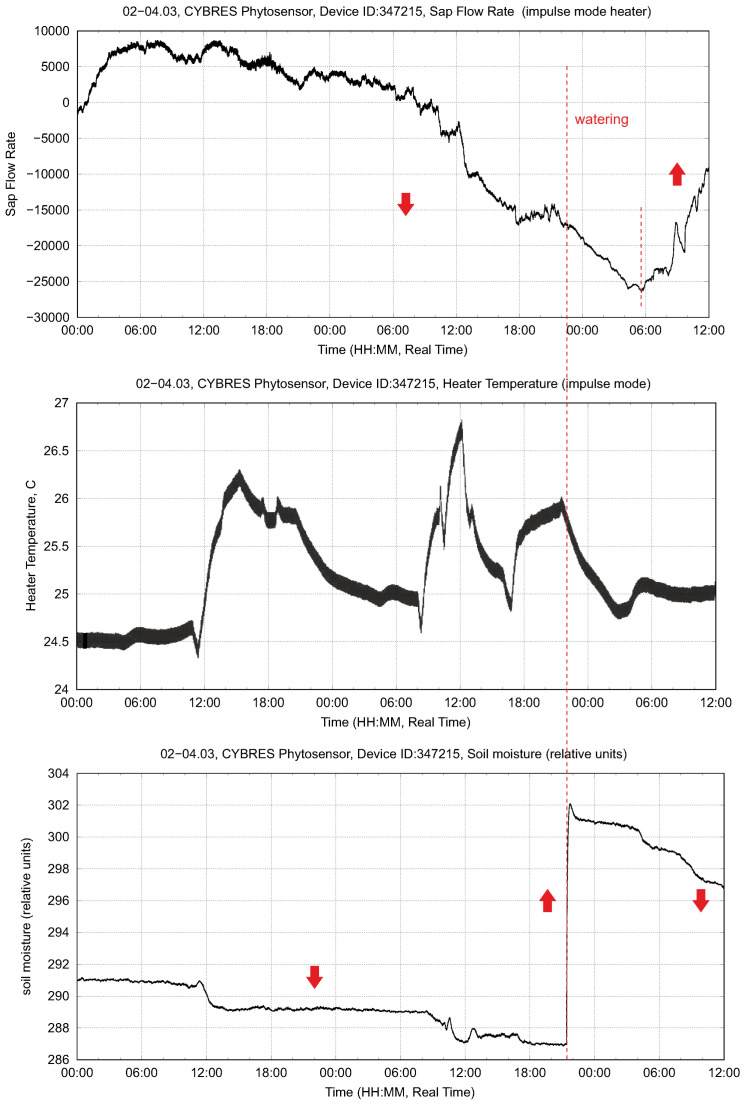
Example of the sap flow dynamics (irrigation stimuli) measured by the heat impulse method: (**upper**) sap flow rate, (**middle**) heater temperature and (**lower**) soil moisture.

**Figure 5:**
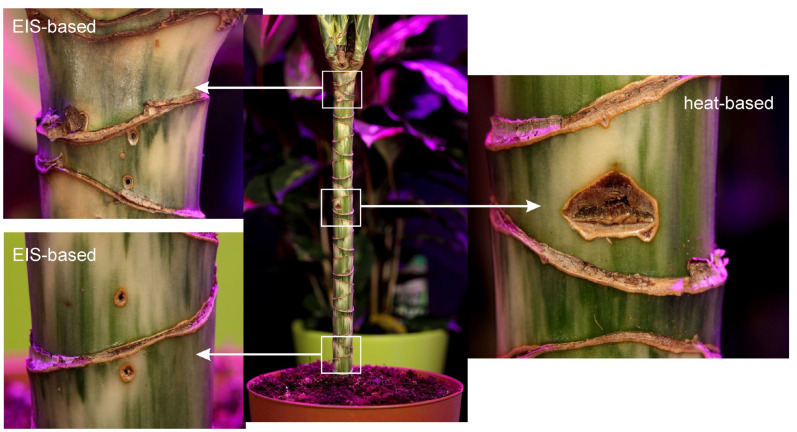
Tissue damage produced by EIS-based (upflow and downflow positions) and heat-based (heat impulse method) sap flow sensors in the setup with the *dracena* plant.

**Figure 6:**
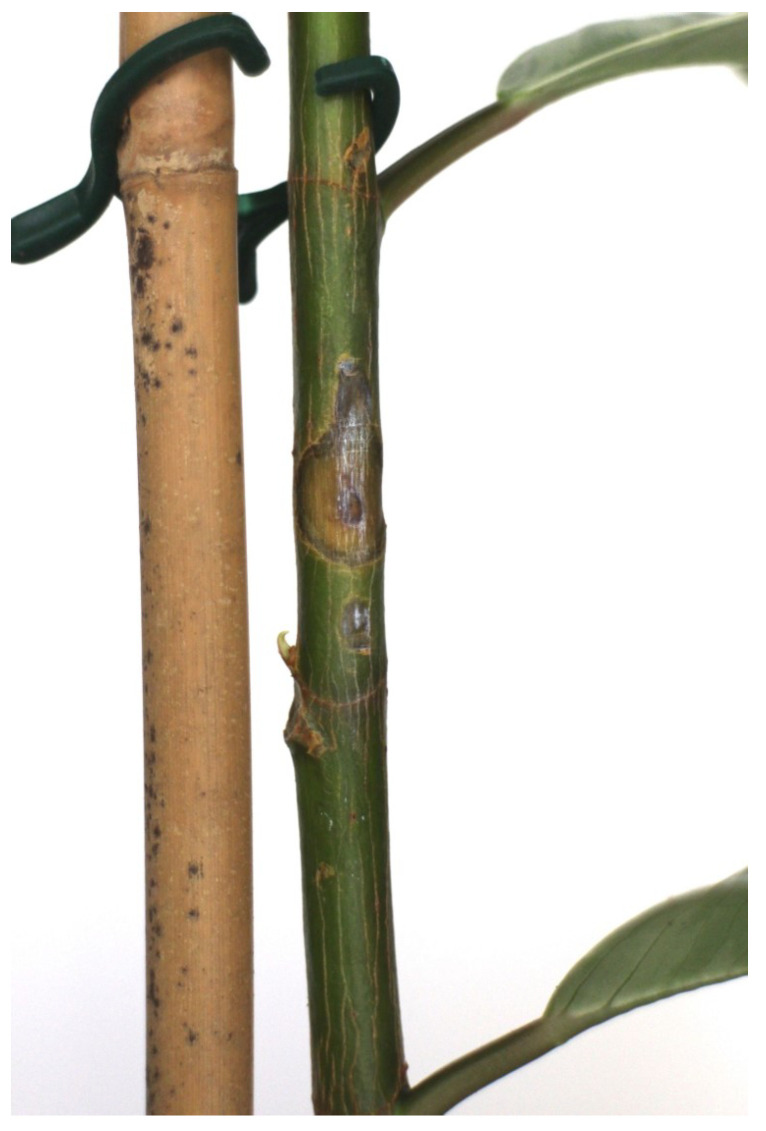
Example of a large amount of tissue damage on a plant with a small stem diameter, caused by a heat-based sap flow sensor.

Heat-based sap flow measurements represent a state-of-the-art method; this is one of main physiological sensors for measuring the movement of water, nutrients and photosynthates throughout a plant. The developed system measured the $∆T_{upper}$ and $∆T_{lower}$ produced by both the heat balance and heat impulse approaches; an example of these measurements is shown in Figure 4. However, the heat-based sensor represents an invasive measurement approach that causes tissue damage if used over a long period of time. Tissue damage depends on the softness of the tissue; woody tissues are less susceptible to damage. Several particular cases are shown in Figure 5; in the soft tissues we observe a large removal of phloem and xylem from the stem, as seen in Figure 6. The heater operates at 1–2 °C higher than the room temperature with a ±1 °C variation. This reaction is not related to overheating but represents a systemic response upon energy influx to the vascular tissue. Another disadvantage of this sensor is that several plant species (e.g., strawberry and microgreens) are generally not suitable for heat-based measurements due to their tiny stems. Moreover, the heater in the sensor is typically a 2 Watt or 5 Watt resistor, which cannot be used for long-term battery operation due to its large power consumption.

As the experiments progressed, we switched to electrochemical measurements using two Ag99 needles inserted into upper and lower stem regions; see Section 3.3. The intensity of the electrical excitation required for impedance spectroscopy seems to have a moderate influence at lower ranges. For example, we did not observe any differences in tissue damage between excitations of 0.01 V, 0.1 V and 1 V. In general, during long-term operation (on the week level), electrochemical methods produce less damage than heat-based methods. Therefore, for practical reasons, we decided to investigate the electrochemical approach in more detail.

### 3.2. Fluid Dynamics in the Stem, Measured by the Electrochemical Method

**Figure 7:**
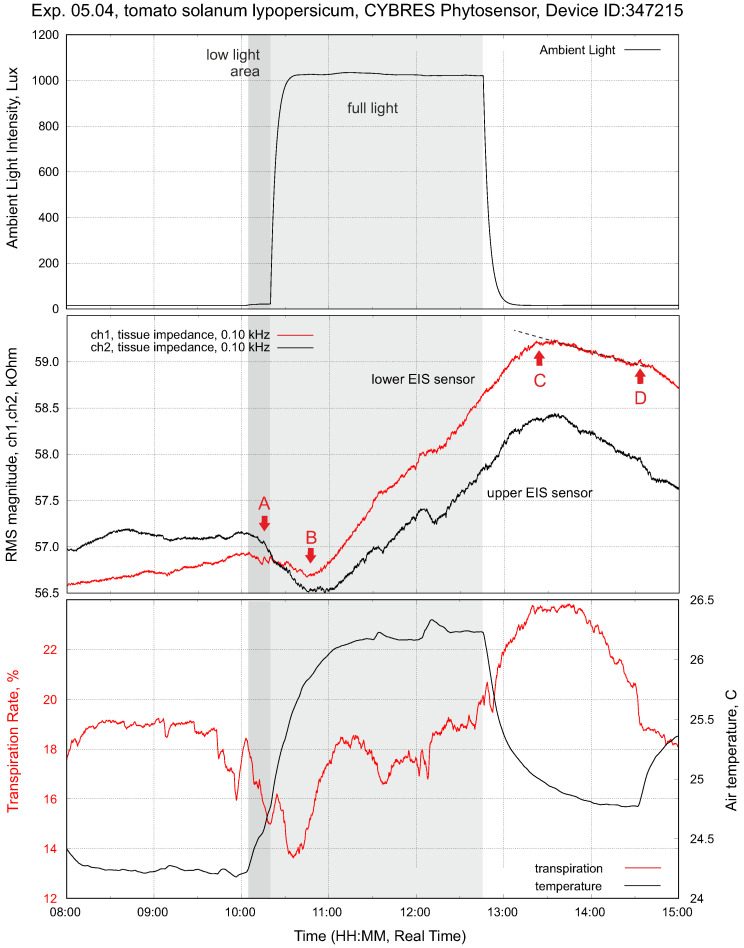
Setup with tomatoes *solanum lypopersicum* (right plant). This is the typical response of tissue impedance (EIS sensor) and transpiration to a light impulse (drip irrigation before measurements; constant decreasing of soil moisture during measurements). The points A and C represent a reaction of tissue impedance on the turning on/off of the phytolight. The points B and D show the change of the impedance trend inside/outside the light-on period; all four points are different for the upper and lower EIS sensors, see further discussion in Section 3.5.

A typical example of impedance measurements and light stimuli with constant soil moisture is shown in Figure 7. In the dark phase (light off), tissue impedance slowly varies according to the internal rhythmic activities of a plant. As soon as the light is on, this triggers the osmotic water movement mechanism (the so-called root pressure [27]) and the ionic fluid content increases in the lower and then upper parts of the stem. This first leads to a decrease in impedance (more ionic fluids). This first phase has a duration from several minutes to 1–2 h. At the same time, leaf transpiration starts to evaporate water; this enables the adhesion-based mechanism that reduces the fluid content in the stem and increases impedance to occur. This is the second phase of impedance dynamics, which is different for the upper and lower sensors.

**Figure 8:**
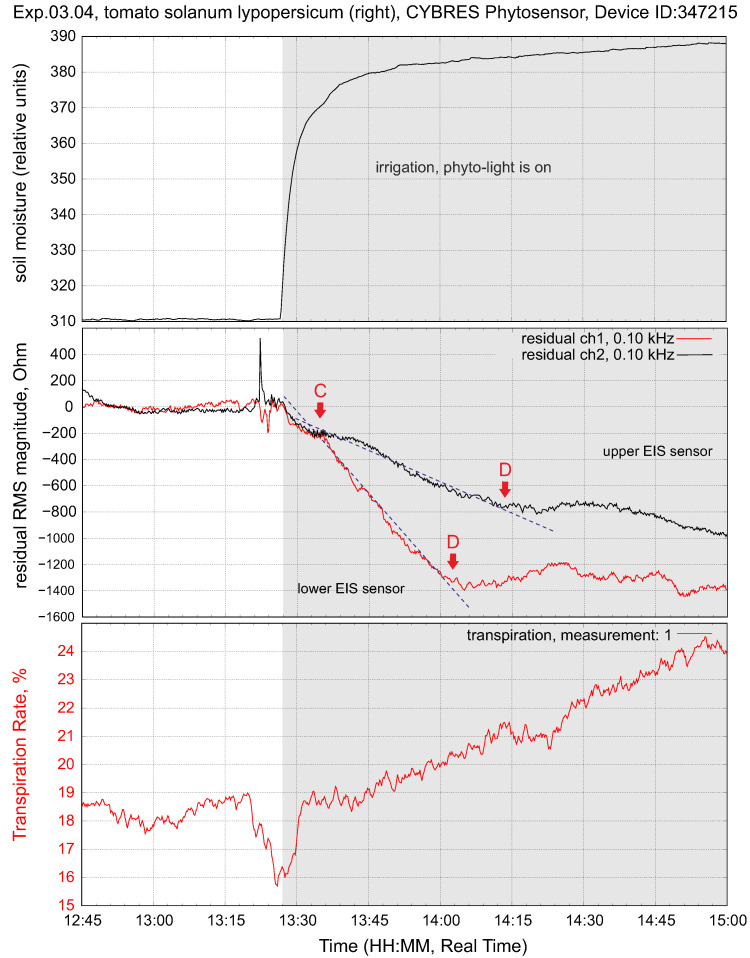
Typical response of tissue impedance and transpiration upon irrigation at constant light flux.

**Figure 9:**
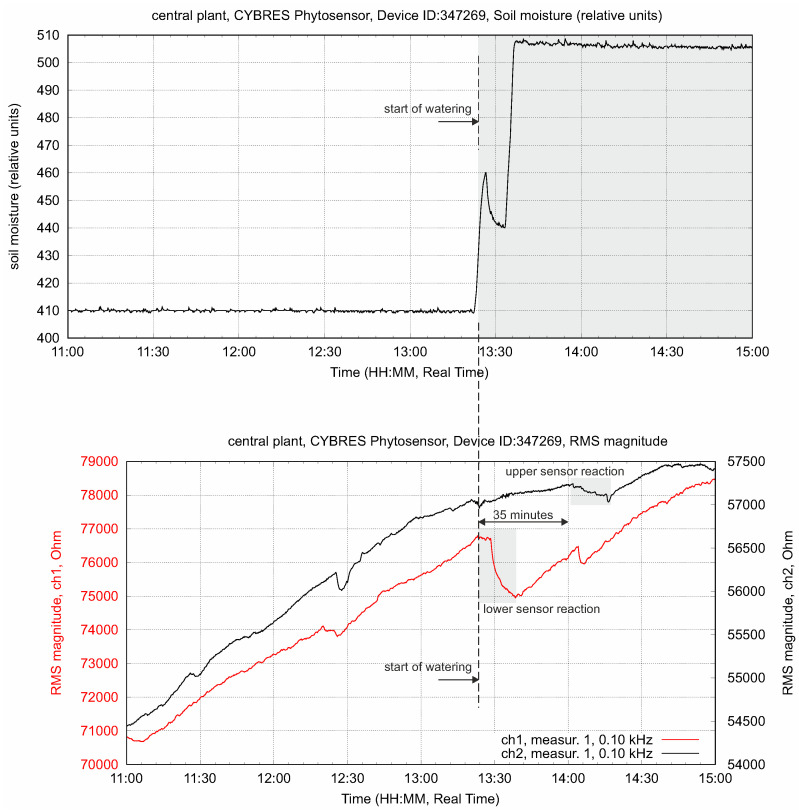
The delay in the propagation response between lower and upper sensors and variation of its intensity. This value indicates the movement of water from roots to leaves under given conditions.

Changing the soil moisture at constant light flux leads to increasing fluid content in the stem. This is shown in Figure 8; as expected, we observe a higher value at the lower EIS sensor. The transpiration sensor reacts to the slow increase in the transpiration rate. Typically, the lower EIS sensor has a greater impedance response to a higher fluid enrichment; see Figure 9. The response delay between the lower and higher sensors, as well as the variation in intensity, can be measured, taking into account the distance between the sensors, and expressed as a numerical value. This value indicates the enrichment of tissues with ionic fluids from the root to leaves under given conditions.

The sap flow dynamics (no light, only one watering stimulus) based on the heat impulse method are shown in Figure 4. One important parameter is the temperature of the heater, since it defines the tissue damages observed after the operation of this sensor. The variations are about ±1 °C. The largest achieved temperature is 26.7 °C with a room temperature of about 24 °C. In the drying phase, the sap dynamics indicate a decay without recognizable daily cycles of sap flow. After irrigation at 21.00, the sap flow increased with the next daily cycle at 6.00.

Comparing the data from electrochemical and heat-based sensors, we observe a similar dynamics related to light and irrigation stimuli. Light and watering increase the sap flow, taking into account daily cycles. However, electrochemical sensors provide more information about fluid movements due to the higher resolution of their measurements. For instance, short-term changes in the fluid transportation dynamics are clearly observed, which are caused by osmotic and transpiration mechanisms.

Uncertainties in electrochemical measurements are related to several factors: homogeneity considerations (see the discussion in Section 5), electrochemical noise, variations in the fluid content due to metabolic activities, inhomogeneous irrigation or the environment. Electrochemical noise provides information about molecular dynamics and can be analysed in several ways, as shown in [28]; for example, it can be used for fast impedance spectroscopy [29]. However, this topic is beyond the scope of this paper, which mainly uses a regression analysis. Variations of fluid content can be understood as a variation of the mean of EIS circadian rhythms in long-term measurements. Analysing these variations may represent a source of additional physiological information; the proposed LRM analysis in Section 3.5 focuses primarily on 24 h diurnal dynamics, discarding long-term changes.

### 3.3. Electrochemical Analysis of Upflow and Downflow Fluids

The difference between the upflow and downflow lies in their content of nutrients and photosynthates, which generate different ionic dynamics [30]. Considering the classic chemical reaction of photosynthesis

(1) $$6{\mathrm{CO}}_{2}+12H_{2}O\overset{hv}{\to }C_{6}H_{12}O_{6}+6O_{2}+6H_{2}O,$$

we see that the upflow in xylem tissue is represented by the LHS of (1), with H_2_O containing different dissolved ions. Among them, potassium (K) and magnesium (Mg) play an important role in ionic dynamics since plants take up both minerals only in their ionic form (as Mg^2+^ and K^+^) [31]. Upflow also transports NO_3_^−^, PO_4_^3−^ and SO_4_^2−^ ions and different ionic solutions of copper Cu and phosphorus P, such as Cu^2+^, H_2_PO_4_^−^ and others [32]. The RHS of (1) represents a downflow in phloem tissue, which contains fewer dissolved ionic molecules due to the decrease in H_2_O and presence of non-ionic sucrose C_6_H_12_O_6_.

Since EIS electrodes penetrate all xylem and phloem tissues, electrochemical measurements always contain a combination of upflow and downflow. In a similar way, thermal sap flow measurements also represent a combination of upflow and downflow. However, different ionic dynamics are detectable by EIS without the functionalization of electrodes. Here, we need to remember that the EIS measurements described in Section 3.1 and Section 3.2 have been performed in the time domain, i.e., measuring the dynamics of impedances over time at a fixed excitation frequency (typically 100 Hz or 450 Hz). To detect different ionic dynamics, we first need to perform measurements in the frequency domain, i.e., measuring changes in the impedances over excitation frequencies [20].

**Figure 10:**
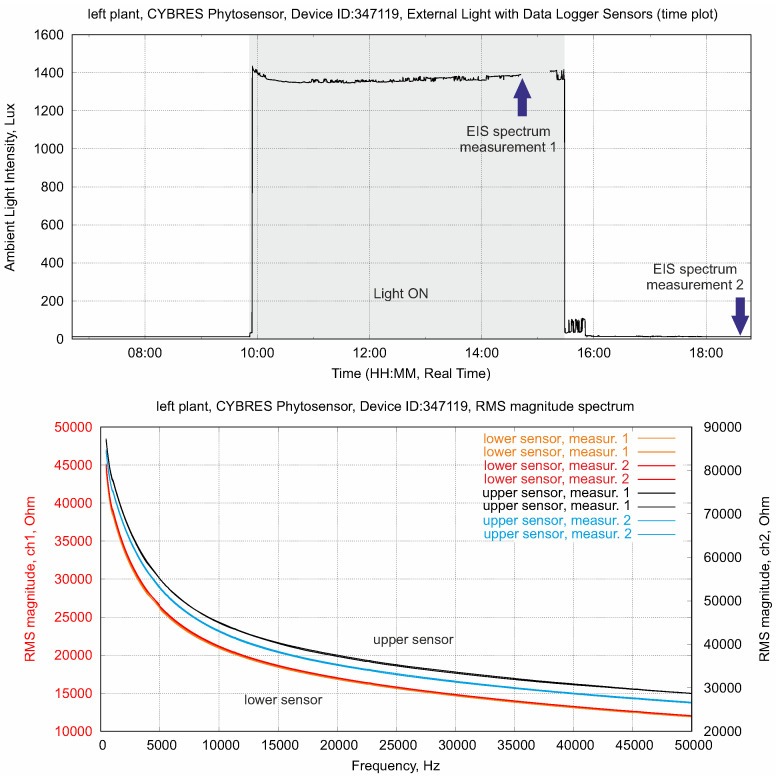
EIS in the frequency domain, applied to the lower and upper sensors. The electrochemical spectrogram of the upper sensor demonstrates clear differences before and after a light excitation (presence of photosynthates in the upper area of stem), whereas the lower sensor has the same ionic content before and after the light excitation (water and nutrients from soil).

This measurement is shown in Figure 10. Spectral EIS is performed twice in the light-on (with photosynthesis) and in the light-off conditions (without photosynthesis) for the lower and upper electrodes. The electrochemical spectrogram of the upper sensor demonstrates clear differences before and after a light excitation (via the presence of photosynthates in the upper area of stem). It is important that the second measurement in the light-off phase demonstrates a lower impedance, as this points to a higher ionic content according to (1). The lower sensor also measures differences between the light-on and light-off phases; however, these differences are much smaller than those in the upper sensor. This reflects the different combination of ionic components and photosynthates in the plant’s sap in the leaf and root areas of the stem. Thus, in vivo impedance spectroscopy allows for the discrimination between aqueous solutions containing high levels of ionic nutrients and low levels of ionic photosynthetic products in a hydrodynamic system.

The result shown in Figure 10 can be also found in EIS in the time domain—typically, it appears in the 30–40 min transition period immediately after the phytolight is turned on or off.

### 3.4. Hydrodynamic Model

**Figure 11:**
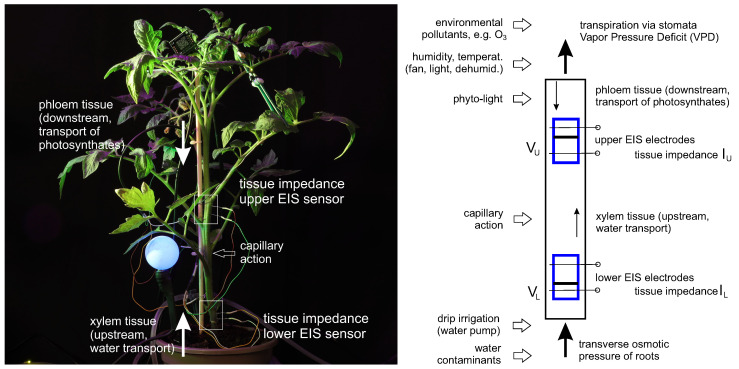
Hydrodynamic model of two-point electrochemical measurements in the upper $V_{U}$ and lower $V_{L}$ stem areas; see description in text.

The EIS measurements shown in the previous sections can be used to develop a holistic hydrodynamic model, which summarizes aspects typical for two-point electrochemical measurements. The effective measurement range of two needles (in one sensor position) is limited by their applied excitation potential, electric field, the dielectric properties of tissues and can be represented as a volume *V* containing fluids; see Figure 11. EIS needles penetrate all tissues, including the phloem and xylem layers, and can be considered as sensors that measure the enrichment of tissues with ionic fluids in a volume *V*. The more fluid the tissue contains, the lower its impedance.

The upper and lower EIS sensors in the stem have two such volumes: $V_{U}$ and $V_{L}$. Water with nutrients is pumped from the roots through transverse osmotic pressure and evaporated through the leaves via the stomata by following the vapor pressure deficit (VPD). In the simplest terms, the fluids evaporated by leaf transpiration go through the volume $V_{U}$ and the fluids obtained from the roots go through the volume $V_{L}$. Due to its low transport velocity and the structure of phloem and xylem tissues, the stem can be considered a sponge-like vertical media with a slow refilling between $V_{U}$ and $V_{L}$. For instance, Figure 9 demonstrates a differential response upon irrigation between its lower and upper sensors that, for a particular setup, is about 35 min.

This slow refilling means that changes in the upper part of the stem (variations in VPD, environmental influences such as ozone-induced stomatal sluggishness [33,34], changes in photosynthetic activities) will be first reflected in $V_{U}$, while changes in the lower part (irrigation, soil moisture, nutrients, water contaminants) will be first reflected in $V_{L}$.

The dynamics of the upper and lower EIS sensors shown in Figure 8 and Figure 9 can be understood in the following way. Turning on the light (point A) starts transpiration and photosynthesis; this decreases the amount of fluids in the upper part of the stem (in the $V_{U}$ area, due to evaporation via the stomata and the photosynthetic reaction (1)). This, in turn, is reflected in the increasing impedance of $I_{U}$. The production of photosynthates introduces the first transient period (the region A–B) until the downflow is stabilized along the whole stem. Any intrinsic or extrinsic variations in VDP, photosynthesis or air pollutants are reflected in the dynamics of the upper EIS sensor—it closely follows the transpiration data.

Turning off the light (point C) stops transpiration and photosynthesis; the $V_{U}$ volume slowly refills and its impedance decreases. This introduces a second transient period (the region C–D) until the downflow is stabilized again. Any intrinsic or extrinsic variations in irrigation, osmotic pressure or water contaminants are reflected in the dynamics of the $V_{L}$ volume and data from $I_{L}$.

The differential dynamics of $V_{U}$ and $V_{L}$ are of special interest. First of all, plant-common events, such as irrigation, first provide more fluids to $V_{L}$ and then, with some delay, to $V_{U}$. Similarly, turning off the light is first reflected in $V_{U}$ and then, with some delay, in $V_{L}$. In all such cases, we observe a delayed reaction between $I_{U}$ and $I_{L}$. The parameters that influence the chemical reactivity and capillary force (approaches from physical chemistry; see more in [15,35]) are measurable in the differential dynamics of $I_{U}$ and $I_{L}$.

Following the physical nature $∆T_{upper}-∆T_{lower}$ of thermal sap flow measurements [36], see Section 3.1, electrochemical $I_{U}-I_{L}$ data have a similar meaning for the region U−L of the stem. However, the difference is that thermal sap flow measurements are conducted in a small region of stem, whereas electrochemical data are gathered from the much larger *U* and *L* regions. Based on discussions about the sap flow rate and sap flux density [37,38], the temporal dynamics $I_{U}^{∆t}$ and $I_{L}^{∆t}$ correspond to the sap flow at *U* and *L*; their differential value $I_{U}-I_{L}$ corresponds to the sap flux in the region U−L. However, the results of thermal and electrochemical measurements do not completely coincide due to their different physical principles.

As mentioned in Section 3.3, EIS in spectral mode can measure ionic content and differentiate between low-/high-ionic fluids such as nutrients and photosynthates. This opens up additional possibilities for data analysis; however, it also requires switching between different measurement modes (spectral and temporal data cannot be obtained from each other).

### 3.5. Characterization of EIS-Based Dynamics in the Hydrodynamic Model

For practical applications of the hydrodynamic model, we need a numerical characterization of EIS data. The following approach describes one of the possible methods for characterization that has been tested on several applications.

**Figure 12:**
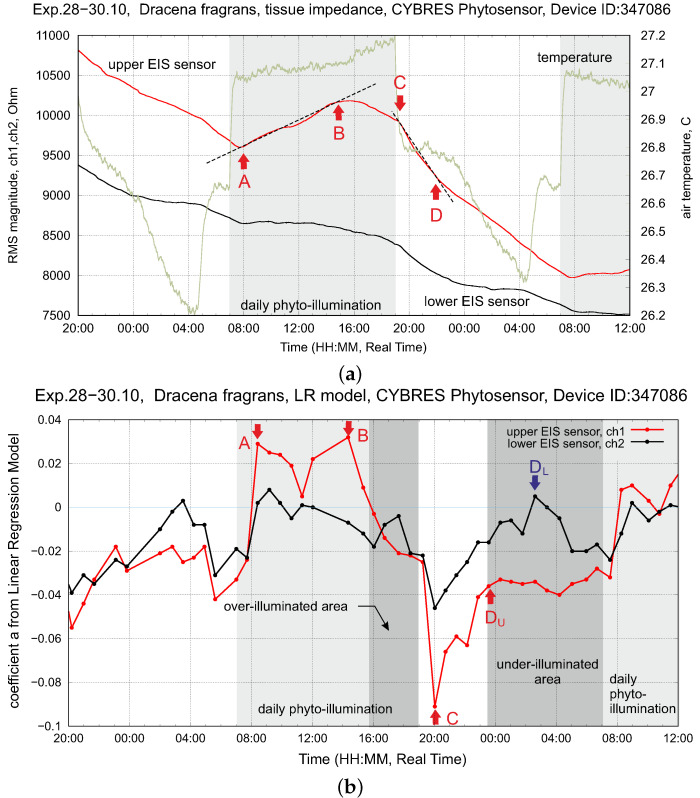
Setup with *dracena fragrans* (left plant). (**a**) Dynamics of tissue impedance (upper and lower EIS sensors) and environmental temperature at constant air movement (fan-off mode) and stable soil moisture (drip irrigation before measurements, constant decreasing of soil moisture during measurements). (**b**) Dynamics of upper EIS sensor over 60 h, shown are the A, B, C, D points used for controlling the PPFD of the phytolight and exploring the WUE-PPFD dependency; see their description in the text.

The dynamics of fluids in the $V_{U}$ and $V_{L}$ areas is represented by the slope of EIS curves; see the points A, B, C, D in Figure 12a, which are well repeated in the circadian rhythms of plants. Considering these points, a characterization of the hydrodynamic model can be achieved by a piece-wise linear regression model (LRM). This approach is formalized as follows. The original data data(x) from EIS sensors are approximated by a linear function with the coefficients a,b:

(2) fit(x)=ax+b

using the Levenberg–Marquardt algorithm [39], where we calculate the residual curve

(3) res(x)=data(x)−fit(x).

For res(x), the rolling window standard deviation $\sigma_{r}$ is calculated as follows:

(4) $$\sigma_{r}=stDev\left(res\left(x\right)\right).$$

Each new i-sample $res(x_{i})$ is analyzed if the following condition is satisfied

(5) $$res\left(x_{i}\right)>3\sigma_{r},$$

a new rolling window starts for the calculation of the rolling standard deviation $\sigma_{r}$ (the Nσ threshold is parametrized in experiments). The LRM coefficient *a* and the time stamp of the i-sample are stored. In this way, the EIS dynamics are represented by a series of coefficients *a* from (2); see Figure 12b. Due to the large computational load, steps (2)–(5) are implemented as an algorithm in Python with highly efficient numerical libraries like NumPy for real-time execution at each data sampling step. LRM coefficients represent an efficient method to determine the optimal time points for changing phytolight and irrigation; see applications of this approach in the following section.

## 4. Applications of Biofeedback-Based Control

### 4.1. Biofeedback-Based Control of Phytolight

Phytolight optimization in greenhouses and hydroponic production is typically based on the light saturation point [40], which is related to the photosynthetic photon flux density (PPFD). There are multiple works that correlate the hydraulic conductance of the soil–root–shoot–leaf pathway with photosynthetic activities [41] and water use efficiency (WUE) [42]. WUE is defined as the biomass yield in unit of plant transpiration and represents how plants convert water into carbohydrates [43,44]. Climatic control based on sap flow [45] suggests that WUE depends on the complex dynamics of sap flow, net radiation, air temperature, wind speed and relative humidity. Maximum WUE is achieved at a specific PPFD value and decreases with a further increase in PPFD. Since PPFD is defined as the number of photons incident per unit time on a unit surface, the variation of on/off times may represent an effective strategy for the control of a phytolight.

Considering Figure 12, we see that $V_{U}$ starts to fill with fluids earlier than the phytolight was off (at about 16.00 every day; the phytolight was turned off at 19.00 every day). This dynamic is repeated by transpiration as well and indicates that photosynthetic activities decrease following the intrinsic metabolic rules of the plant (e.g., a low level of fluids in the stem). This area can be counted as an inefficient region of WUE with a non-efficient usage of PPFD and can be detected by

(6) $$light==ON\;\;\;and\;\;\;a_{i}^{U}<0$$

which leads to the ‘phytolight off’ actuation.

In the light-off area, we observe a similar dynamic, where $V_{U}$ is first actively filled up with fluids (the point $D_{U}$ in Figure 12b), after which this slope is more or less constant as $(a_{i-1}-a_{i})\approx 0$ or $a_{i}\approx 0$. Considering data from the low EIS sensor, we observe a characteristic peak (the point $D_{L}$ in Figure 12b) corresponding to the maximal refilling from $V_{L}$ to upper stem areas. After $D_{L}$, the stem is fully refilled with fluids. Here, the argument follows the logic that the maximum WUE is under the conditions

(7) $$light==OFF\;\;\;and\;\;\;max(a_{i}^{L}),$$

where $max(a_{i}^{L})$ is found in rolling windows with past values. Following the water deficit strategy, any point between $D_{U}$ and $D_{L}$ can be used for the ‘phytolight on’ actuation. Conditions (6) and (7) can be used to control far-red light (or any other spectral light, e.g., blue light) and balance between 12:12 and 16:8 on–off rules for the phytolight [46].

**Figure 13:**
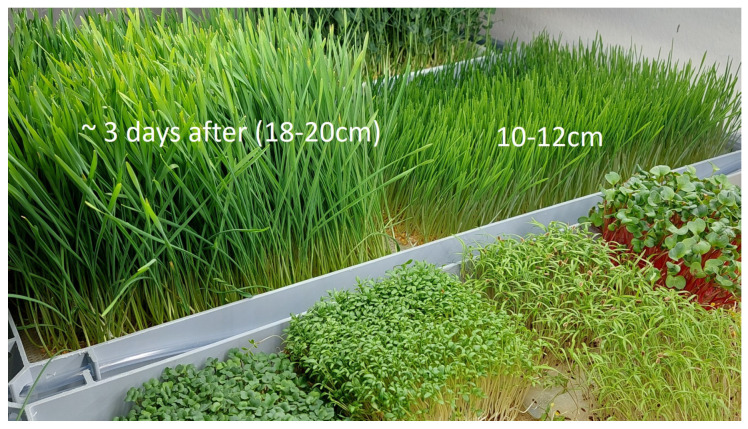

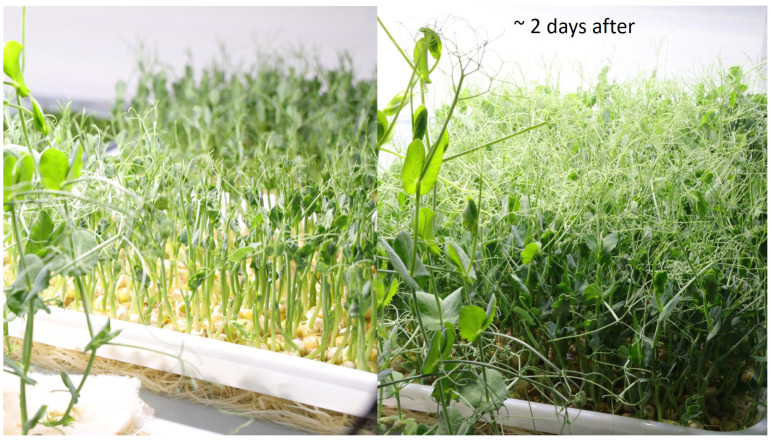
The microgreen production setup, with wheat and peas for testing the biofeedback-based control of phytolight.

Rules based on Conditions (6) and (7) are introduced in the Python algorithm from Section 3.5, which calculates LRM coefficients for the hydrodynamics model and controls phytolight/irrigation in real time. This approach was tested for microgreen production; see Figure 13. By varying the $D_{U}$ and $D_{L}$ points and recognizing different B and C points, we implemented several strategies for (1) minimizing energy consumption, (2) minimizing the growth time or (3) maximizing biomass production. Strategies (2) and (3) represent the same approach, which, however, was used for different productive species: for instance, wheatgrass production minimizes the growth time, whereas pea production maximizes the biomass. The most simple case is energy optimization, where this dynamical approach provides about 25–30% energy optimization in relation to the 16:8 fixed rule, especially for early stages of microgreen production. The two other strategies are more complex; here, the ratio between the on and off periods does not essentially change, but instead of one on period we created several on periods with different spectral lights, which allowed us to change the vertical farm from a 7-day-cycle to a 4–5-day one, to reduce the wheatgrass cycle from 10 days to 7–8 days. Keeping the same cycle in pea production allowed us to reach about 30% more biomass (in combination with biofeedback-based irrigation, see next section) in our tested cases.

### 4.2. Biofeedback-Based Irrigation

Biofeedback-based irrigation represents a different approach than phytolight, since most of the water is lost through transpiration and we have to consider additional parameters such as the vapor pressure deficit [47], the degree of aeration, temperature variations caused by LED light, and others.

**Figure 14:**
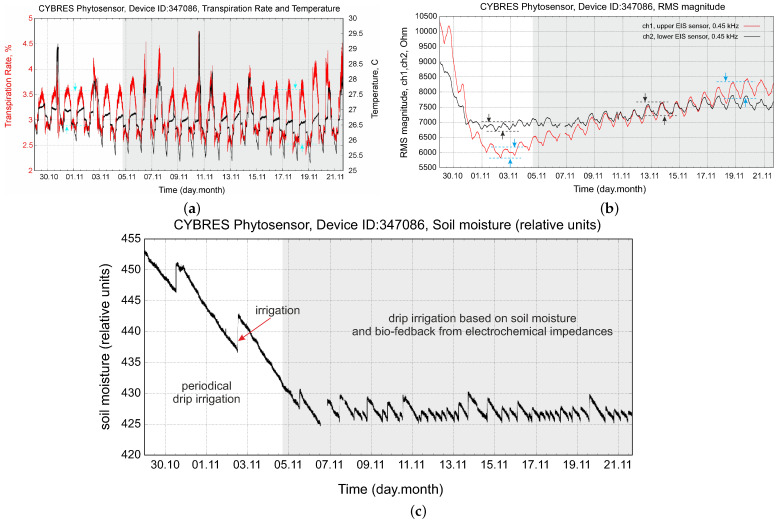
Example of periodical and biofeedback-based irrigation: (**a**) transpiration and temperature data; (**b**) tissue impedances measured by $I_{L}$ and $I_{U}$ sensors; (**c**) soil moisture with two different irrigation strategies.

Periodical irrigation is the simplest method; however, it leads to non-efficient water consumption. We show this case in Figure 14. Periodical irrigation produces essential hydrodynamic variations in the $I_{L}$ and $I_{U}$ sensors and can increase or decrease the water enrichment of tissues depending on the VPD; see the left part of Figure 14b. More advanced approaches are based on moisture measurements, allowing us to keep a constant soil moisture, and on exploring water stress with a controlled water deficit strategy, supplementary irrigation and several other techniques [48,49]. The right part of Figure 14c demonstrates irrigation based on soil moisture (which determines the switch-on point) and the EIS amplitude of circadian rhythms (which determines the duration of drip irrigation). We see that this approach allows us to keep the amplitude of circadian rhythms and the absolute values of the electrochemical impedances almost constant.

**Figure 15:**
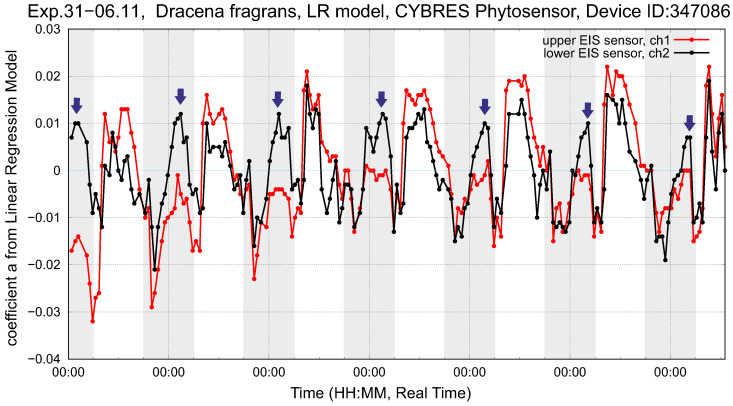
Setup with *dracena fragrans* (left plant). Shown are the LRM coefficients of (2) for the upper and lower EIS sensors during the six days of the experiment. Night periods are marked by a grey bar, and the arrows indicate maximum water consumption.

**Figure 16:**
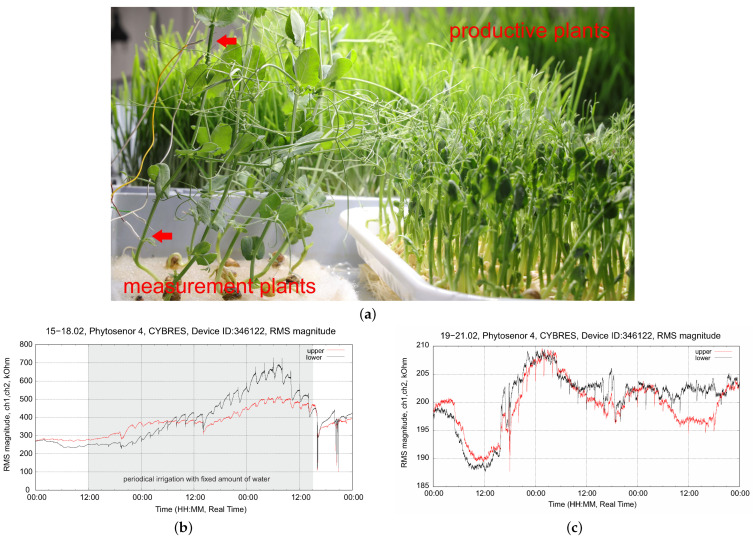
Biofeedback-based irrigation considering the refilling dynamics of the $I_{L}$ sensor for growth without substrate/soil: (**a**) measurements using EIS electrodes in microgreen (pea) production; (**b**) example of periodical irrigation strategy; (**c**) periodical irrigation strategy with biofeedback.

Considering data from the $I_{L}$ sensor, we observe periods when the $V_{L}$ volume is actively filled up with water from the roots and stable periods with a slow variation of $V_{L}$. Considering soil as another sponge-like volume, the refilling between soil–root and root–$V_{L}$ volumes leads to temporal irregularities in water consumption that can be minimized by applying irrigation during these periods. Figure 15 demonstrates the dynamics of LRM coefficients, where the effect of fast water refilling on the dynamics of the $I_{L}$ sensor is shown by arrows. To compensate for high water consumption, additional irrigation can be switched on at these moments. This approach is of particular interest for soil/substrate-free cultivation; an example of such an irrigation strategy is shown in Figure 16 for pea production.

Growth without substrates, typical for wheat and pea in vertical farms, requires frequent and periodic irrigation. Figure 16b shows this case with periodic irrigation every 2 h with a fixed amount of water. We observe increasing peaks in the dynamics of the $V_{L}$ sensor, which represent development processes in young plants. However, we also see that in some cases, plants are over- or under-watered; moreover, the $V_{U}$ sensor almost does not respond during periodic irrigation, which indicates an insufficient duration of the irrigation pulses. In Figure 16c, we changed the strategy to biofeedback, considering the refilling dynamics of the $I_{L}$ sensor, as shown in Figure 15. The system controls the duration of the irrigation pulses (which still occur every 2 h). We see that the effect of over-irrigation is eliminated and, moreover, water enrichment is achieved throughout the stem (and not only in the lower part). This example also illustrates the separation between measurement and production plants that is sought in a vertical farm containing microgreens; see Figure 16a. In fact, only EIS sensors with thin electrodes can be used in such cases.

### 4.3. Stress Detection: Water Deficit and Ozone

Nonspecific stress detection is conducted by analyzing hydrodynamic deviations from a normal circadian rhythm recorded in standard (without stress) conditions. Stress-driven physiological changes in the water transportation system are measurable long before they become visible in the plant’s morphology, degradation of leaves or other visual observations. An EIS-based approach enables early stress detection; we demonstrate this using two examples.

**Figure 17:**
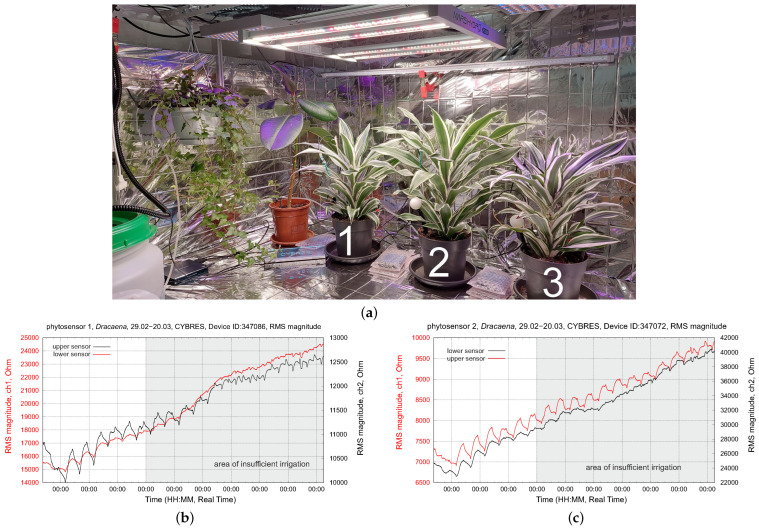
Testing water-deficit stress with a WDS: (**a**) The setup, with 3 *dracena fragrans* plants with an applied WDS; (**b**,**c**) EIS dynamics of the upper and lower EIS sensors during 3 weeks of measurements. All plants have different temperature conditions, leading to different VPDs.

The first example originates from the water deficit strategy (WDS) discussed in the previous section. A WDS requires the continuous monitoring of physiological state, otherwise it can lead to degradation of the plant organism. The WDS was tested over three weeks using three plants, as shown in Figure 17a. All plants had a similar initial state but different temperature conditions, leading to different VPDs. The EIS dynamics of plants 1 and 2 are shown in Figure 17b,c.

These plants had the highest daily temperature (directly under the LED lamp, without aeration) and thus the highest water loss. By the middle of the tested period, the dynamics of their upper $I_{U}$ sensors become very flat (no water movement in the upper part of stem); about two weeks after this test, the leaves of both plants became dry and dark yellow. Finally, both plants died. Plant 3 had a weak but still recognizable circadian rhythm and survived the test with several damaged leaves. Based on the EIS circadian dynamics in Figure 17b,c, the WDS for plants 1 and 2 should be changed during the second week of the test to avoid the degradation of their water transportation system.

**Figure 18:**
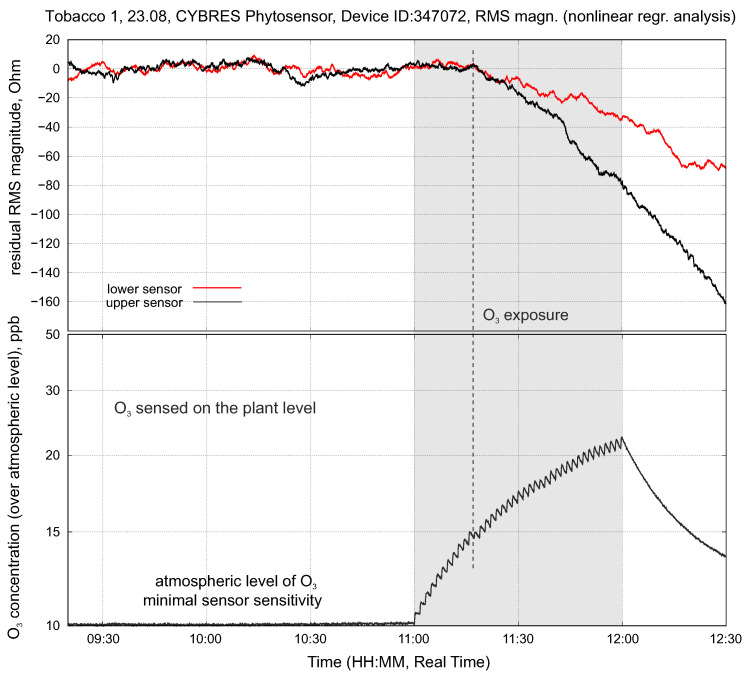
Testing O_3_ stress: an example of the physiological response in the upper and lower EIS sensor positions when exposed to a low concentration of O_3_.

The second example relates to environmental monitoring, and in particular to biological O_3_ detection, which is one of the most major environmental pollutants [50]. Ozone triggers various reactions in plant organisms, in particular, it affects stomatal regulation [51]. Since changes in stomata conductance induced by O_3_ in turn alter the hydrodynamic parameters of water–sap transport in the plant stem, the biological detection of ozone can be conducted by sensing electrochemical tissue impedances in situ [22]. Figure 18 demonstrates the response of a tobacco plant to ozone exposure. We applied a regression analysis to the data obtained from the upper and lower sensors, as described in Section 3.5. The O_3_ stress closes the stomata and increases the water content in the stem. This deviation is measurable with EIS as a decreasing impedance mainly in the upper and then in the lower part of the stem. A separate work is devoted to this topic [52].

## 5. Discussion

In this discussion, we will cover three topics that arise during practical implementations: advanced AI-based control schemes, homogeneity considerations and the reliability of EIS sensors.

The control schemes for phytolight or irrigation shown in previous sections are a simple reactive approach based on biofeedback using electrochemical data. More complex control strategies require advanced numerical and statistical analyses, as well as AI applications, to work with phytosensor data [9]. In such cases, we need to take into account multiple physiological parameters of the plant, soil and environment, as well as the previous state of the actuating system. Some of these parameters may conflict with the main objective and require multi-parametric optimization. An example is a homeostatic control that switches phyto-LEDs on and off based on biofeedback. In this case, we used the action potential measured from a plant (this example was first introduced in the ‘Flora Robotica’ project, funded by the European Commission, grant ID: 640959) [7]. Since the action potential responds very fast to a changing environment and follows the physiological cycles of the plant, its variations can underline adaptive strategies for controlling light. An important consideration is that LEDs increase environmental temperature, so we also need to monitor light and temperature and consider how long the system has been in a dark state.

**Figure 19:**
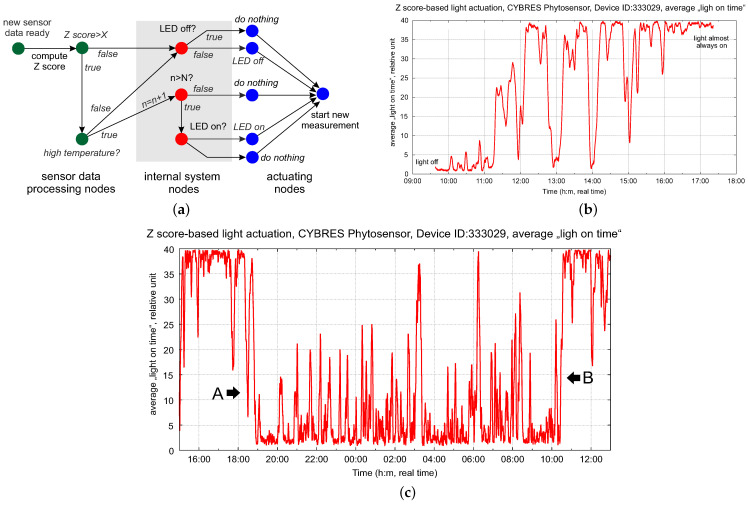
(**a**) Logic of the control scheme for the adaptive control of light based on the action potential, with event-driven Petri nets; (**b**) dynamics of the average ‘light on’ time (in relative units) with biofeedback; (**c**) three days after the start of using the light with biofeedback, the plant demonstrates adaption of its ‘on’ and ‘off’ times (boundaries A and B), which do not coincide with day and night periods.

The logic of this control scheme is shown in Figure 19a; we use the Z-score calculation for biopotentials. When implementing such a logic to control the phyto-LEDs, one needs to consider the internal states of the actuators (in which actual state is the actuator) and of the system (how long has the system been in its dark state). Such diagrams are known as event-driven Petri nets, where the token represents the current state of the system traveling in the network. This is discussed in more detail in [25]. This control scheme can be understood as an algorithm with *if-then* Conditions (6) and (7) embedded in the topology of its information processing network. The event-driven approach allows for complex exploration of biohybrid systems, considering their hidden biological states.

The dynamics of the average ‘light on’ time are shown in Figure 19b; the phytolight using biofeedback is continuously turning on and off (very similar to PWM modulation controlled by the plant). Selecting the threshold for the Z-score requires some manual tuning and can be improved with an adaptive strategy, e.g., with reinforcement learning. However, we discovered an interesting effect after several days of using the phytolight with biofeedback—the boundaries of the A and B regions (‘on’ and ‘off’ times, see Figure 19c) stabilize despite the non-optical selection of the threshold. In addition, they do not coincide with the day and night periods and develop their own adaptive photoperiodic rhythm.

The result of this experiment is similar to the adaptive strategy with an artificial LED light already discussed in Section 4.1—hydrodynamic rhythms divide the lighting period of 18:8 or 12:12 into several periods that do not coincide with natural day/night rhythms. Here, we touch on the adaptation mechanisms of circadian clocks [23] and more generally on the abilities of plants to self-adapt and self-learn [24]—these important topics obviously go beyond the scope of this article. We mention them in the discussion to show that there is a potential way to combine biohybrid systems and AI research, with promising outcomes. We also see several business opportunities for developing dedicated control strategies for different plant species using the same (phyto)sensing/actuating system.

Another topic of discussion is homogeneity considerations, e.g., as shown in Figure 17a—how well do several measuring plants represent many productive plants? Using dedicated measuring plants only is typical for electrophysiological measurements and can be applied to leafy greens, microgreens, and other similar types of plant. The plants selected for measurements met several requirements for their developmental stage and were intact, but, in general, the measurement data from multiple plants were in good agreement. Such plants can be used for a longer period of time (e.g., microgreens can be harvested every week; measuring plants can be used for measurements in the same facility for several weeks). The same approach can be applied to plant roots (see [10,11]), especially in the production of wheatgrass.

Comparing the heat-based method and EIS, we see several advantages to sensors based on electrochemical impedances. They are simpler and therefore more reliable in indoor and outdoor applications. Electrochemical spectroscopy can distinguish several fluid types inside the stem. Due to their high hardware requirements, only one heat-based sensor is feasible at a reasonable cost. Impedance sensors can increase the number of channels by using a multiplexer. We see a clear advantage of having at least two sensors in the upflow and downflow positions; increasing the number of channels allows for more accurate measurements.

## 6. Conclusions

The main focus of this work was on biohybrid vegetable systems with phytosensors measuring electrochemical impedances or performing impedance spectroscopy in vivo and in situ. The underlying model takes into account the hydrodynamics of the fluid transport system, verified by sap flow data from heat-based sensors, irrigation and light stimuli, circadian rhythms, stress and other factors. From a practical point of view, two-sensor measurements satisfy most practical schemes for spectral phytolight control, adaptive irrigation or stress detection. An interesting feature of the EIS performed in vivo is the ability of electrophysiological sensors to distinguish high- and low-ionic aqueous solutions using nutrients and the products of photosynthesis in the plant stem. Tests conducted indoors and outdoors showed the high reliability of EIS sensors.

We demonstrated a closed-loop control of various phytoactuators based on real-time biofeedback in vertical farming, environmental monitoring and biohybrid AI research. Agricultural applications are especially relevant; here, energy optimization can reach 25% to 30% compared to non-optimized 16:8 lighting. The biological monitoring of air pollution is another important topic; we have dedicated a separate publication to this use case of phytosensors. Open points relate to the water properties of hydrodynamic systems at the physicochemical level and aquaporin channels, which can also be measured using electrochemical methods. These approaches, as well as their combination with mobile NMR sensors, represent topics for future work.

## Figures and Tables

**Table 1:** Overview of measured (electro)physiological and environmental parameters/stimuli.

Physiological Parameter	Description
tissue impedance	4× Ag99 electrodes, 1V excitation, time–frequency EIS
electrochemical spectroscopy	time–frequency EIS, fast EIS for in situ sap analysis
biopotentials	4× Ag99 electrodes, input impedance $10^{-15}$ Ohm, input bias current ±70 pA
leaf transpiration	differential air–humidity-based method, CYBRES
leaf temperature	precision LM35 sensor
thermal sap flow	heat balance and heat pulse methods, 3× *t*-sensing, PID stabilized, CYBRES
fluid content of tissue	(electrochemical) 4× electrode method, CYBRES
**Sensed Environm. Param.**	**Description**
light, humidity, temperature	APDS-9008-020, HIH-5031-001, LM35CA
EM emission, magnetometer	450 Mhz–2.5 Ghz RF power meter, MAX2204 chip; 3-axis, LIS3MDL
soil humidity, temperature	capacitive-based sensor, CYBRES
water parameters	conductivity, pH, temperature, etc.
CO_2_, PM1-2.5-10, O_3_	SCD4x, accuracy ±(40 ppm + 5%); SPS30, accuracy 10%, CENSIRION
**Environmental Stimuli**	**Description**
light, irrigation, temperature	full-spectrum light, IR/UV supplementary light, automatic irrigation system, heater
EM emission, O_3_, PMx	mobile phones (GSM 890-1.805 MHz) WIFI routers (2.4 GHz), weak magnetic fields and O_3_ generators

## Data Availability

The datasets presented in this article are not readily available because of an ongoing study. Requests to access the datasets should be directed to the author.
